# Lean non-alcoholic fatty liver disease (Lean-NAFLD) and the development of metabolic syndrome: a retrospective study

**DOI:** 10.1038/s41598-022-14701-0

**Published:** 2022-06-29

**Authors:** Wenting Wang, Jianping Ren, Wenzhao Zhou, Jinyu Huang, Guomin Wu, Fenfang Yang, Shuang Yuan, Juan Fang, Jing Liu, Yao Jin, Haiyang Qi, Yuyang Miao, Yanna Le, Cenhong Ge, Xiantao Qiu, JinJing Wang, Ping Huang, Zixin Liu, Sheng Wang

**Affiliations:** 1 School of Public Health, Hangzhou Normal University, No.2318 Yuhangtang Road, Hangzhou, 311121 Zhejiang Province China; 2 Zhejiang Laboratory for Systems & Precision Medicine, Zhejiang University Medical Center, 1369 West Wenyi Road, Hangzhou, 311121 Zhejiang Province China; 3 Affiliated Hangzhou First People’s Hospital, Zhejiang University School of Medicine, No.261 Huansha Road, Hangzhou, 310006 Zhejiang Province China; 4 School of Nursing, Hangzhou Normal University, No.2318 Yuhangtang Road, Hangzhou, 311121 Zhejiang Province China; 5 Department of Biology and Chemistry, Zhejiang Institute of Metrology, No.300 Xiasha Road, Hangzhou, 310018 Zhejiang Province China; 6 Department of Hepatology, Affiliated Hospital of Hangzhou Normal University, Normal University, HangzhouHangzhou, 310015 Zhejiang Province China; 7 Institute of Hepatology and Metabolic Diseases, Hangzhou Normal University, Hangzhou, 311121 Zhejiang Province China; 8 Hangzhou Medical Association, No.70 Kaiyuan Road, Hangzhou, 310006 Zhejiang Province China

**Keywords:** Non-alcoholic fatty liver disease, Metabolic syndrome

## Abstract

Lean NAFLD is a special phenotypic closely correlated with metabolic syndrome (MS). The aim of this study is to investigate the MS development and the gender differences in lean NAFLD population. Participants were divided into 4 groups by BMI and NAFLD status. Descriptive analysis was performed to characterize baseline information. A total of 18,395 subjects were participated, and 1524 incident cases of MS were documented. Then, Kaplan–Meier curves were used to present the MS outcomes in different groups, and the NAFLD was found to be a riskier factor than obesity for MS. Subgroup analysis showed significantly higher MS incidence in female than male among lean NAFLD group, which is different from other groups. Although with higher prevalence in male, lean NAFLD seems to be a more harmful phenotype for females according to the TG, ALT and GGT levels. The logistic regressive analysis was performed to show the impact of NAFLD status and BMI changes on MS risk. Lean non-NAFLD subjects merely developed to NAFLD with no BMI status changes exhibited highest MS risk (ORs = 1.879, 95% CI 1.610–2.292) than that with both BMI increase and NAFLD development (ORs = 1.669, 95% CI 1.325–2.104). It also suggests the metabolic specificity of this population.

## Introduction

Nonalcoholic fatty liver disease (NAFLD) is one of the most common chronic liver disease^1,2^, and also an emerging risk factor for type 2 diabetes mellitus, cardiovascular disease, renal diseases and all-cause mortality^3^. The prevalence of NAFLD is reported to be approximately 25% worldwide^4^, ranges from 24.0 to 46.0% in western industrialized countries and from 7.9 to 54.0% in Asian countries^5,6^. As obesity is a major risk of NAFLD, the prevalence of NAFLD is expected to continue rising paralleled with the global epidemic of obesity^6,7^,especially in China where obesity has increased at an estimated annual rate of 0.32%.

Indeed, the classical phenotype of NAFLD patients occur in obese or overweight^8^ (body mass index (BMI) > 25 kg/m^2^), there exists a substantial proportion of individuals with NAFLD have a BMI below 25 kg/m^2^ or even below 23 kg/m^2^ which usually referred to as no-obese or lean NAFLD^9,10^. According to the latest research, the global prevalence of lean and non-obese NAFLD was approximately 5.1% and 12.1%^11^, respectively, and the lean NAFLD is commonly found in Asia. Although having similar complications with obese NAFLD, lean NAFLD is increasingly viewed as a significant phenotype, which showed different risk for these complications like type 2 diabetes (T2D) and cardiovascular disease (CVD)^12,13^.

In recent years, accumulating evidence have demonstrated that lean NAFLD is a progressive condition, and patients with lean NAFLD exhibited some features of MS^14,15^, and might have worse outcomes than obese counterparts. To date, the relationship between lean NAFLD and MS is still not fully understood, and more long-term studies are required to fill in the research gap. In addition, most studies showed that lean NAFLD is highly relevant to males^16^, but lack attention to characteristics in females, and the gender differences of lean NAFLD are also not clearly investigated. Hence, we performed this study in a large Chinese population to show the association of lean NAFLD and the MS development, and to find out the difference between males and females with lean NAFLD.

## Results

### Characteristics of participants in the baseline

A total of 18,395 participants in the baseline (10,393 males and 8002 females, aged 44.92 ± 13.80 years old) were recorded. Based on the categories of BMI and NAFLD status, 8951, 3911, 1034 and 4499 participants were classified into four groups: lean non-NAFLD group, obesity/overweight non-NAFLD, lean NAFLD, and obesity/overweight NAFLD, respectively. The baseline characteristics of all participants are listed in Table 1. As can be seen, the differences among the four groups were statistically significant in regarding to age, gender, waist circumference, blood pressure and most biochemical indicators except the neutrophil percentage. For lean NAFLD participants, the levels of blood pressure, FPG, HGB, HbA1c, TG, LDL-C, RBC, WBC, ALT, GGT, TP, ALB, urea and UA were lower than that in obesity/overweight NAFLD subjects, but higher than that in obesity/overweight and lean non-NAFLD subjects. It worth noting that fructosamine, TC, PLT and ALP present highest levels in lean NAFLD subjects. Further LSD test was carried out for pairwise comparison, and the result showed that the differences of PLT and ALP between Lean NAFLD and overweight/obese NAFLD group were indistinctive (*p* = 0.389 and 0.481, respectively), while the fructosamine and TC still showed significance (*p* = 0.002 and 0.032, respectively).

**Table 1:** Baseline demographic and biochemical characteristics of participants.

	Non-NAFLD (N = 12,862)		NAFLD (N = 5533)		*p* value
	Lean	Obesity/overweight	Lean	Obesity/overweight	
Participants, n (%)	8951(48.66%)	3911(21.26%)	1034(5.62%)	4499 (24.46%)	
Age(yr)	**42.13 ± 13.35**	**45.34 ± 13.99**	**50.66 ± 13.15**	**48.80 ± 13.28**	< 0.001
Male	3382	2669	675	3667	< 0.001
Female	5569	1242	359	832	
BMI (kg/m^2^)	20.47 ± 1.59	24.65 ± 1.4	21.83 ± 1.01	26.16 ± 2.26	< 0.001
WC (cm)	74.94 ± 6.92	86.05 ± 7.12	82.05 ± 6.85	91.95 ± 7.61	< 0.001
SBP (mmHg)	71.28 ± 9.54	75.82 ± 9.76	76.38 ± 9.35	79.48 ± 9.72	< 0.001
DBP (mmHg)	114.09 ± 15.28	123.43 ± 15.01	123.8 ± 15.71	128.64 ± 14.97	< 0.001
FPG (mmol/L)	4.89 ± 0.6	5.03 ± 0.72	5.23 ± 1.04	5.24 ± 0.94	< 0.001
Fructosamine (mmol/L)	**1.48 ± 0.19**	**1.49 ± 0.19**	**1.58 ± 0.23**	**1.55 ± 0.21**	** < 0.001**
HGB(g/L)	137.77 ± 15.25	145.70 ± 15.22	146.38 ± 14.49	150.60 ± 12.81	< 0.001
HbA1c (%)-	5.42 ± 0.53	5.50 ± 0.61	5.65 ± 0.78	5.70 ± 0.74	< 0.001
TG (mmol/L)	1.07 ± 0.59	1.37 ± 1.02	1.85 ± 1.27	1.92 ± 1.22	< 0.001
TC (mmol/L)	**4.67 ± 0.86**	**4.80 ± 0.88**	**5.07 ± 0.94**	**5.01 ± 0.93**	** < 0.001**
HDL-C (mmol/L)	1.52 ± 0.35	1.34 ± 0.31	1.30 ± 0.32	1.20 ± 0.28	< 0.001
LDL-C (mmol/L)	2.44 ± 0.65	2.70 ± 0.67	2.90 ± 0.71	2.96 ± 0.7	< 0.001
RBC (10^9^/L)	4.61 ± 0.45	4.84 ± 0.47	4.86 ± 0.48	4.97 ± 0.43	< 0.001
WBC (10^9^/L)	5.81 ± 1.47	6.16 ± 1.66	6.20 ± 1.48	6.58 ± 1.55	< 0.001
PLT (10^9^/L)	**215.61 ± 51.9**	**213.9 ± 49.73**	**221.19 ± 53.73**	**219.67 ± 51.11**	** < 0.001**
NEUT (%)	57.15 ± 8.13	56.98 ± 7.84	56.77 ± 7.72	56.76 ± 7.51	0.264
ALT(U/L)	18.2 ± 24.48	23.34 ± 28.94	28.12 ± 44.51	34.02 ± 30.32	< 0.001
ALP(U/L)	**63.83 ± 16.65**	**68.62 ± 16.66**	**72.79 ± 17.66**	**72.38 ± 16.76**	< 0.001
GGT(U/L)	18.99 ± 14.6	26.52 ± 23.54	33.34 ± 34.7	38.42 ± 34.09	< 0.001
TP(g/L)	74.72 ± 3.56	74.63 ± 3.56	74.81 ± 3.52	75.07 ± 3.42	< 0.001
ALB(g/L)	46.43 ± 2.46	46.3 ± 2.5	46.64 ± 2.36	46.66 ± 2.36	< 0.001
Urea(mmol/L)	4.63 ± 1.18	4.89 ± 1.27	4.91 ± 1.14	5.02 ± 1.19	< 0.001
UA (μmoI/L)	293.84 ± 72.63	341.07 ± 79.96	351.14 ± 76.56	380.54 ± 80.32	< 0.001
Creatinine (μmoI/L)	75.97 ± 12.21	83.03 ± 14.85	80.70 ± 11.94	85.07 ± 11.96	< 0.001

Bold font: Factors showed significant difference between lean and obesity/overweight NAFLD subjects via pairwise comparison.

### Risk of MS in different BMI and NAFLD status

**Figure 1:**
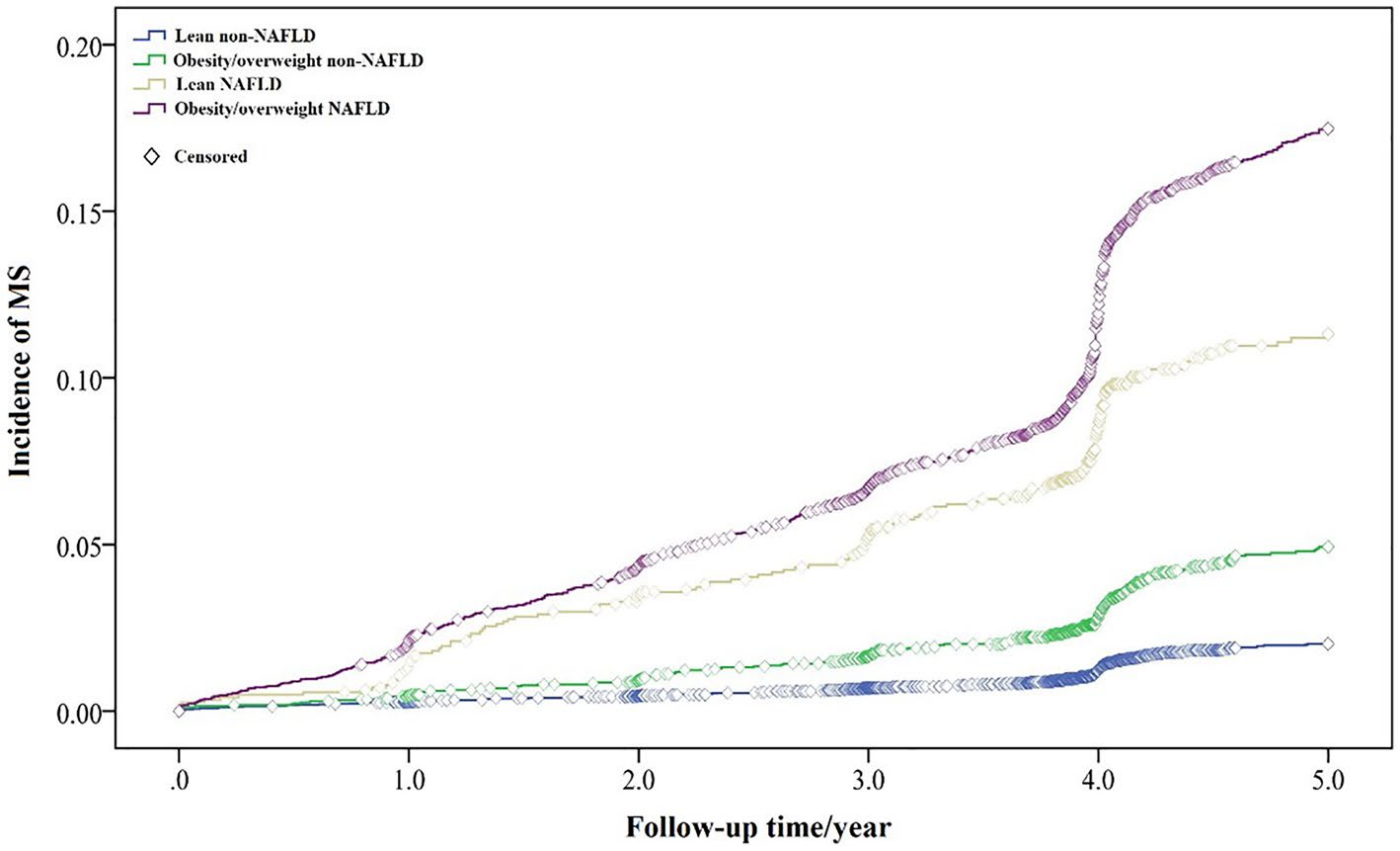
Kaplan–Meier curves of MS events stratified by BMI and NAFLD status.

**Figure 2:**
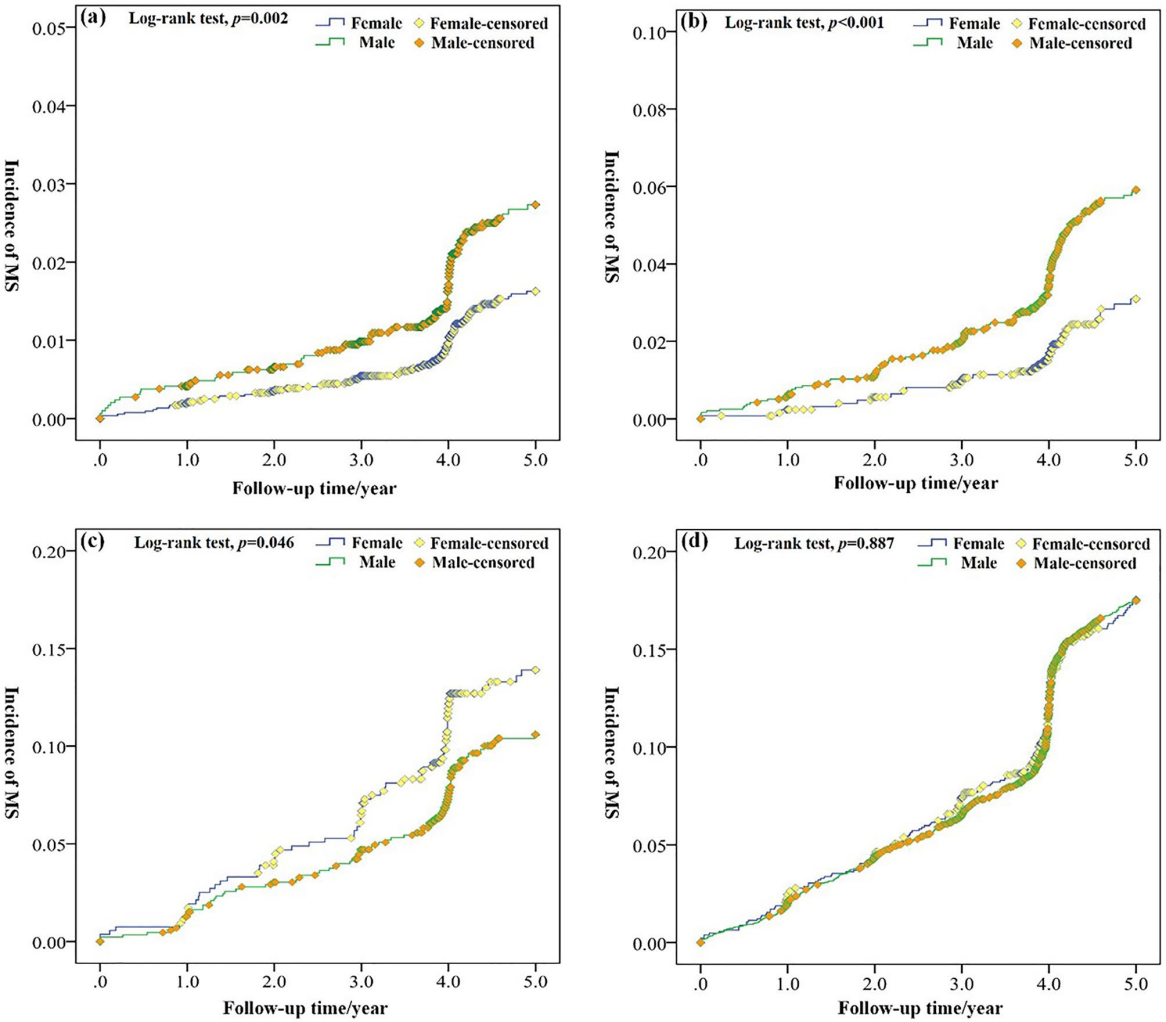
The incidence of MS in different gender. (**a**) lean non-NAFLD, (**b**) obesity/overweight non-NAFLD, (**c**) lean NAFLD, (**d**) obesity/overweight NAFLD.

During the search process, 1524 incident cases of MS were documented. With a median follow-up time of 4.42 years, 94,781.5 person-year was obtained. The Kaplan–Meier curves for endpoints in participants with different BMI and NAFLD status were shown in Fig. 1. The cumulative incidence of MS in lean NAFLD subjects is higher than obesity/overweight non-NAFLD subjects, and lower than obesity/overweight NAFLD subjects. A subgroup analysis in gender showed that the cumulative incidence of MS is significantly higher in female than male in lean NAFLD group (Fig. 2c). By contrast, male subjects showed higher cumulative incidence in lean and obesity/overweight non-NAFLD groups (Fig. 2a,b). For overweight/obese NAFLD participants, the difference of MS incidence is not significant in gender (Fig. 2d, *p* = 0.887 > 0.05).

Compared with the lean non-NAFLD group, the multivariate-adjusted hazard ratio (HR) of MS in obesity/overweight non-NAFLD, lean NAFLD and obesity/overweight NAFLD groups were 2.04 (95% CI 1.53–2.72), 3.17 (95% CI 2.37–4.25), and 5.48 (95% CI 4.33–6.93), respectively. As can be seen, the risk of MS in lean NAFLD group is higher than that in obesity/overweight non-NAFLD group. Subgroup analysis for MS in four groups was also carried out, respectively. As is listed in Table 2, the fructosamine showed highest risk in every group compared to other factors. For lean NAFLD group, RBC and UA were also risk factors of MS with HR > 1, and UA only showed significance in lean NAFLD group. HGB and PLT were consider as protective factors with HR < 1.

**Table 2:** Subgroup analysis of the HR for MS in four groups.

Risk factor		Lean non-NAFLD		*p*	Obesity/overweight non-NAFLD		*p*	Lean NAFLD		*p*	Obesity/overweight NAFLD		*p*
		HR	95%CI		HR	95%CI		HR	95%CI		HR	95%CI	
Gender	Female	1	–	–	1	–	–	1	–	–	1	–	–
	Male	1.617	0.827–3.161	0.160	**2.124**	**1.065–4.238**	**0.033**	0.918	0.442–1.904	0.217	0.924	0.716–1.191	0.541
Age		1.013	0.994–1.033	0.178	**1.026**	**1.007–1.045**	**0.006**	0.996	0.975–1.016	0.676	0.999	0.991–1.007	0.772
Fructosamine		**63.511**	**18.871–213.752**	** < 0.001**	**20.645**	**5.343–79.768**	** < 0.001**	**20.998**	**6.164–71.528**	** < 0.001**	**10.472**	**6.558–16.721**	** < 0.001**
HGB		**0.975**	**0.957–0.994**	**0.012**	**0.967**	**0.946–0.987**	**0.002**	**0.962**	**0.940–0.985**	**0.001**	**0.978**	**0.970–0.986**	** < 0.001**
HbA1c		0.778	0.585–1.034	0.084	0.628	0.418–0.943	0.255	0.925	0.710–1.206	0.566	0.864	0.775–0.962	0.080
TC		0.858	0.716–1.028	0.097	0.894	0.745–1.074	0.231	1.017	0.977–1.058	0.421	0.977	0.932–1.025	0.340
LDL-C		1.314	0.978–1.766	0.070	1.230	0.895–1.690	0.201	0.972	0.908–1.041	0.419	1.048	0.963–1.140	0.275
RBC		**1.059**	**1.003–1.118**	**0.040**	1.011	0.987–1.037	0.376	**1.148**	**1.017–1.296**	**0.026**	**1.169**	**1.116–1.225**	** < 0.001**
WBC		**2.519**	**1.468–4.322**	**0.001**	1.662	0.951–2.904	0.075	1.224	0.693–2.162	0.486	**1.401**	**1.136–1.728**	**0.002**
PLT		1.001	0.997–1.005	0.629	1.000	0.996–1.004	0.965	**0.993**	**0.989–0.998**	**0.003**	**0.996**	**0.995–0.998**	** < 0.001**
NEUT		1.011	0.987–1.036	0.365	1.007	0.982–1.032	0.604	1.007	0.981–1.035	0.588	1.000	0.990–1.010	0.996
ALT		0.985	0.966–1.004	0.124	1.007	0.998–1.016	0.135	1.004	0.991–1.017	0.582	0.998	0.995–1.002	0.452
ALP		**1.008**	**1.002–1.014**	**0.006**	0.998	0.993–1.003	0.421	1.001	0.996–1.006	0.641	1.001	1.000–1.003	0.129
GGT		0.999	0.987–1.011	0.843	1.009	0.998–1.021	0.099	1.005	0.993–1.018	0.385	1.002	0.998–1.006	0.235
TP		**0.934**	**0.873–1.000**	**0.049**	**0.922**	**0.865–0.982**	**0.012**	0.973	0.908–1.043	0.438	**0.949**	**0.926–0.973**	** < 0.001**
ALB		**1.196**	**1.073–1.333**	**0.001**	**1.300**	**1.165–1.452**	** < 0.001**	1.055	0.939–1.186	0.364	**1.194**	**1.145–1.245**	** < 0.001**
Urea		1.026	0.859–1.226	0.773	**0.776**	**0.644–0.935**	**0.008**	0.869	0.722–1.046	0.137	0.962	0.901–1.027	0.244
Creatinine		0.988	0.966–1.011	0.314	1.008	0.989–1.027	0.422	1.001	0.977–1.027	0.921	1.000	0.996–1.004	0.959
UA		1.103	0.999–1.006	0.131	1.101	0.998–1.004	0.389	**1.104**	**1.001–1.007**	**0.006**	1.101	1.000–1.002	0.124

Significant values are in bold.

### Association between BMI, NAFLD status changes and MS risk

Among the 18,395 subjects in the baseline, the BMI and NAFLD status were changed between four groups during the followed-up periods (Fig. S1(a)). The highest proportion of participants were lean non-NAFLD subjects without status changes (32.32%). The transition from obesity/overweight non-NAFLD to lean NAFLD showed lowest proportion (0.31%). About 1.75% of lean non-NAFLD and 1.84% of obesity/overweight NAFLD subjects were changed to lean NAFLD status, respectively, while 1.99% of participants were in lean NAFLD status throughout. The cumulative incidence of MS in participants with status changes was also presented in Fig. S1(b). The lean non-NAFLD and obesity/overweight NAFLD status throughout exhibited lowest and highest MS incidence, respectively.

**Figure 3:**
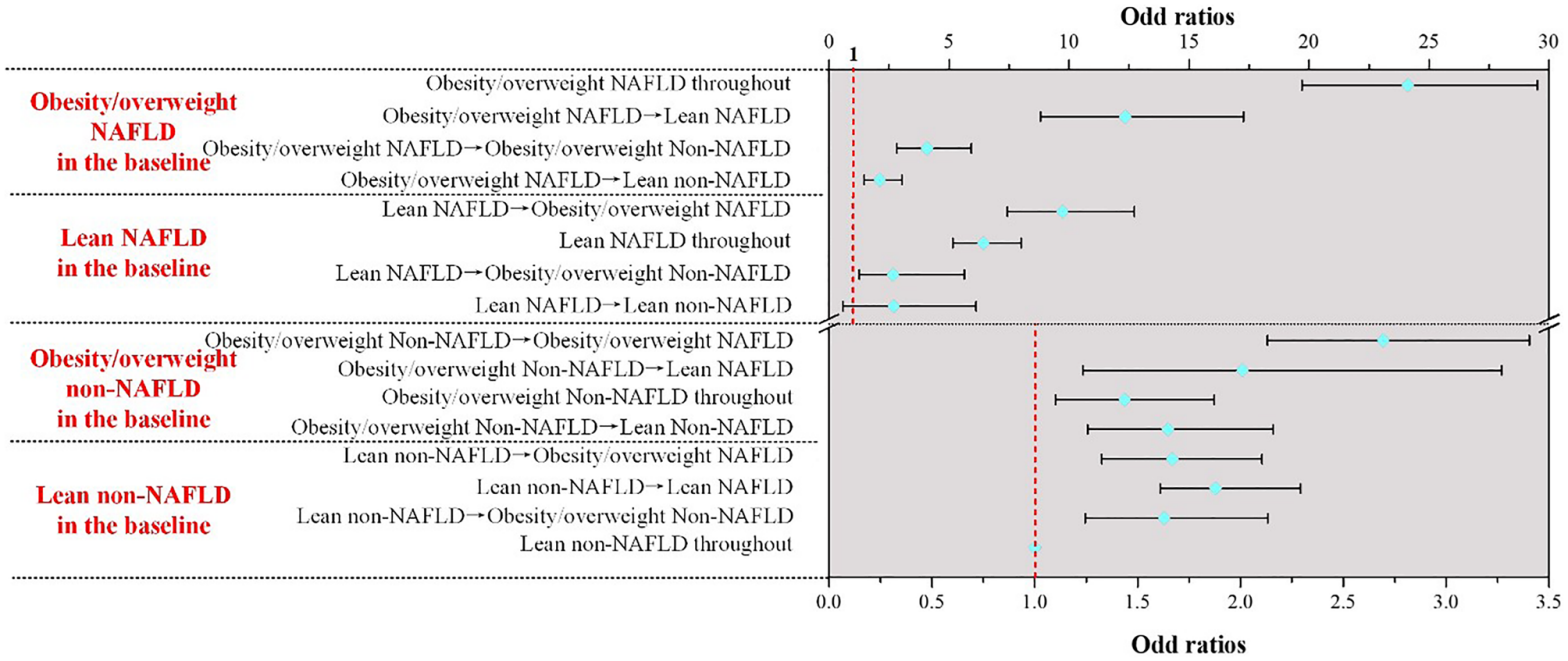
Association between status changes and MS outcomes.

The relationship between the status changes and MS risk was investigated via logistic regression, and all the status changes were significantly related with MS (*p* < 0.05). As is shown in Fig. 3, participants with baseline status of NAFLD presented higher MS risk than that with baseline of non-NAFLD. For all the NAFLD subjects in the baseline, the MS risk raised with the increasing of BMI, and reduced with the recovery of NAFLD. Nevertheless, this rule is not applicable for non-NAFLD subjects in the baseline. In non-NAFLD population, subjects with no status changes throughout present lowest risk of MS among the group with the same baseline (OR = 1 and 1.436 (95% CI 1.101–1.873), respectively). Meanwhile, these developed to NAFLD with no BMI status changes exhibited highest MS risk in both lean and obesity/overweight non-NAFLD population OR = 1.879 (95% CI 1.610–2.292) and 2.694 (95% CI 2.131–3.403), respectively.

## Discussion

Previous studies have shown that Lean-NAFLD is a metabolically unhealthy status with especially high incidence among Asian population, and closely linked with various metabolic diseases including MS^17^ and diabetes^18^. In this study, the baseline demographic characteristics of participants showed that TC present highest levels in lean NAFLD population. Moreover, it exhibited significant difference between lean and overweight/obese NAFLD groups. The similar phenomenon was also observed in Feng et al.’s research^19^. Serum TC is a common risk factor of NAFLD. Recent studies suggested that lean NAFLD subjects always higher TC intake than overweight/obese NAFLD^20,21^, and accompanied by altered cholesterol metabolism^22^. It should be noted that the lower HGB levels in lean NAFLD than overweight/obese NAFLD subjects was observed in our study, and this result is opposite to Akyuz et al.’s research, which showed higher HBG levels in lean NAFLD subjects^23^. The race difference of participants may be the possible reason to induce this result.

In the Fig. 1, the Kaplan–Meier curves showed the higher cumulative incidence of MS in lean NAFLD population than that in obesity/overweight non-NAFLD subjects. In a recent, Hu et al.’s cross-sectional study also found that lean NAFLD present higher MS risk than overweight/obese non-NAFLD^24^. Two possible reasons may account for this phenomenon. One reason is that lean NAFLD is more likely a specific metabolic disease that is stronger correlated with MS than obesity^24^. Another one is the limitation of BMI in reflection of abdominal fat. The abdominal fat, which represented visceral fat accumulation, is an important index of MS. In the development of NAFLD, the excess free fatty acids (FFAs) play a critical role in hepatic steatosis^25^, and FFAs mainly from the visceral adipose tissue. The BMI is not capable of reflecting the distribution of different adipose tissue depots, which is a key factor relevant to the metabolism. In brief, NAFLD showed higher MS risk than obesity. The higher HR of lean NAFLD (HR = 3.17) than obesity/overweight non-NAFLD (HR = 2.04) also confirmed this conclusion.

Subgroup analysis revealed that females are more susceptible to MS than males in lean NAFLD group. It is significantly different from other groups. The incidence of MS is significantly higher for male subjects in non-NAFLD populations, while the difference in gender is not significant for overweight/obese NAFLD. Our result seems to be an indication that lean NAFLD is a gender-specific phenotype. To understand this phenomenon, the baseline data were further grouped by gender (Supplementary materials table S1). As can be seen, most factors present similar trends in four groups between male and female. However, the TG, ALT and GGT levels presented different trends between genders. The TG is increased with BMI and NAFLD severity in males. For female subjects, it exhibited significantly higher levels in lean NAFLD group than other groups. Since TG is a component of MS, the higher TG levels inevitably lead to the higher risk of MS in females. In Leung et al.’s study, high serum triglyceride level is also found as a key factor of lean NAFLD^26^. Due to the relatively higher proportion of females lean NAFLD subjects (44.3%) in their study, the serum TG level is regarded as a core factor of whole lean NAFLD population, although the higher risk of NAFLD activity is also observed in female lean NAFLD subjects than that of males. The ATL and GGT level gives further explanation of this phenomenon. ALT and GGT are liver enzymes that is sensitive to hepatic injury, and large amounts of studies showed that the elevated hepatic enzymes are manifestations of NAFLD and MS. Hyo et al.’s study revealed that serum ALT and GGT concentrations are correlated with the incidence of NAFLD^23^ and MS. ALT is an indicator of liver injury with high specificity, and associated with hepatic IR^27^. The elevated ALT levels also can be used as marker of MS development^28^. Besides, the elevation of GGT is also implicated in the hepatic steatosis and hepatic IR. Among female subjects, the ALT and GGT presented similar or even higher levels in lean NAFLD group compared to overweight/obese NAFLD groups. By contrast, for male subjects, the significantly higher levels of ALT and GGT were observed in overweight/obese NAFLD than that in lean NAFLD group. The ALT and GGT levels showed that females with lean NAFLD suffered from serious liver injury which is equivalent to that of overweight/obese NAFLD population. As TG is the major transport form of FAs that metabolized in liver, the more serious liver injury means that a reduced capability of liver to deal with excessive TG in serum. It is important to note that the average age of lean NAFLD patients is relatively higher than other three groups, which means higher proportion of females at menopause or post-menopause. In previous studies, menopausal status showed significant impact on the MS factors likes TG and LDL cholesterol levels^29^, and post-menopausal status seems to increase the risk of MS^30^. To reduce the influence of this age-related physiological change, in the case of satisfying the minimum sample size, the average age of other three groups were adjusted in accordance with the lean NAFLD females by reducing the sample size, while the gender proportion was not varied with the adjustment. In this condition, the MS incidence is still significantly higher for males in non-NAFLD population, and the difference also showed no significance in gender for obesity/overweight NAFLD subjects (Supplementary material figure S2). According to all the results mentioned above, it can be inferred that lean NAFLD is a more harmful phenotype to females with worse triglyceride metabolism.

The cox regression analysis showed that the MS risk of lean NAFLD is higher than obesity/overweight non-NAFLD, but lower than obesity/overweight NAFLD. In subgroup analysis, fructosamine is the most significant risk factor for MS with 61.511-fold, 19.645-fold, 19.998-fold and 9.472-fold higher risk in four groups, respectively. Besides, UA is a particular risk factor of MS that only showed significance in lean NAFLD group. Fructosamine can be synthesized from the combination of fructose and protein molecules, while the fructose also leads to the rising of UA. Lanaspa et al.’s study demonstrated that hepatic adipogenesis can be regulated by UA through the oxidative stress of mitochondria, and the high UA exposure enhanced the superoxide process, which lead to the steatosis in liver^31^. In Zheng et al.’s study, a positive correlation between UA levels and NAFLD risk in lean subjects was observed^32^. Nakagawa et al.’s research also showed that UA levels is related to the risk of fructose-induced MS and NAFLD^33^.

The changes in BMI and NAFLD status over time have been proved as predictors of MS-related diseases like hypertension and diabetes. Sung et al.’s study showed that NAFLD status changes is associated with development of hypertension, and the transition from non-NAFLD to NAFLD increased risk of hypertension^34^. In Lee et al.’s research, BMI status changes is related to the incidence of diabetes^35^. Thus, exploring the impact of BMI changes and NAFLD development on the risk of MS is of great significance. As a special phenotype, lean NAFLD status changes were focused upon. As can be seen, lean NAFLD and overweight/obesity non-NAFLD are hardly converted to one another (0.59% and 0.31%), which indicates that these two phenotypes were quite different. Based on the logistic regression analysis, it can be observed that subjects developed to NAFLD without BMI status changes showed highest risk in the non-NAFLD population with the same baseline. The transition from lean non-NAFLD to lean NAFLD (ORs = 1.879, 95% CI 1.610–2.292) is even riskier than that changed to obesity/overweight NAFLD (ORs = 1.669, 95% CI 1.325–2.104). This result suggested that lean subjects developed to lean NAFLD refer to a metabolic phenotype which is different from other status transitions.

This study still has several limitations. First, only ultrasonic diagnosis was used for NAFLD detection without confirmation by other methods. As an operator-dependent technique, the accuracy of ultrasonography is limited especially for mild NAFLD. Second, because of the limitation of retrospective study, some possible influence factors like insulin resistance and female menopausal status are unable to be analyzed without relevant data. In addition, this study was only conducted in Zhejiang province, multi-center studies in larger scales are further required to confirm our findings.

In summary, our study found that lean NAFLD is a more harmful phenotype to females which increased their risk of MS. Although with lower prevalence in females, this phenotype may have worse metabolism of serum TG in female population due to the more serious liver damage. The UA level is also observed as a featured factor associated with the risk of MS merely in lean NAFLD subjects. In addition, we found that lean non-NAFLD subjects that merely developed to NAFLD with no BMI status transition represent a special population with higher MS risk than that with both BMI increase and NAFLD development. It is also a demonstration that NAFLD in lean subjects is a distinctive phenotype in metabolism.

## Methods

### Study population

**Figure 4:**
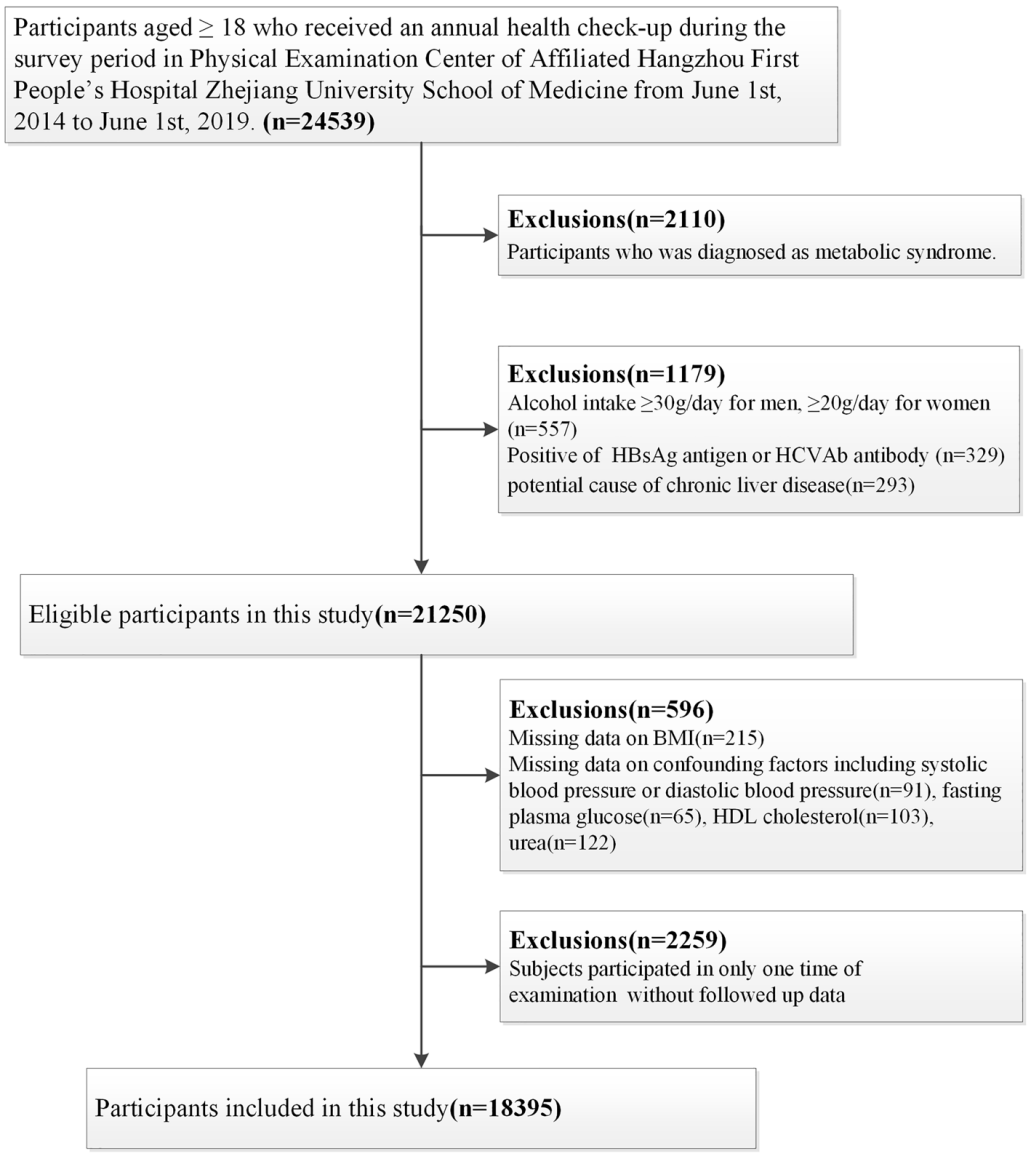
The flowchart of study participants selection.

As is shown in Fig. 4, a population-based retrospective study was conducted in Hangzhou, Zhejiang, China, from June 1st, 2014 to June 1st, 2020. A total of 24,539 participants aged ≥ 18 received an annual health check-up during the survey period in physical examination center of Affiliated Hangzhou First People’s Hospital Zhejiang University School of Medicine. Since our objective is to evaluate the longitudinal association between lean NAFLD and the development of MS, we excluded participants who was diagnosed as MS (n = 2110) according to criteria jointly defined by the International Diabetes Federation (IDF) and AHA/NHLBI^36^.

Among these subjects,1179 of them were excluded who had any of the following condition at baseline:

1. previous/current excessive alcohol intake (≥ 30 g/day for men and ≥ 20 g/day for women);
2. hepatitis B surface antigen (HBsAg) or hepatitis C virus antibody (HCVAb) positively;
3. potential cause of chronic liver disease including autoimmune hepatitis, and use of hepato-toxic drugs.

Afterwards, we excluded 596 participants with missing data on confounding factor (i.e. BMI, systolic blood pressure (SBP) or diastolic blood pressure (DBP), fasting plasma glucose and so on). In the remaining 20,654 subjects, 2259 of them were further excluded due to only one time of examination participated during the study, which is not suitable for further statistical analysis without followed up data. Finally, 18,395 adults constituted our study cohort.

### Ethics

This study was conducted in accordance with the guidelines in the Declaration of Helsinki. The Ethics committee of Affiliated Hangzhou First People’s Hospital Zhejiang University School of Medicine approved the study, and all the participants gave their informed consent.

### Data collection and measurements

The data were collected by qualified medical examiners at each visit, which included measurements of height, weight, waist circumference, blood pressure, etc. BMI was calculated as weight (kg) divided by height (m) squared. Waist circumference was measured at the midpoint of the line between the anterior superior iliac crest and the 12th costal margin in the standing position to the nearest 0.1 cm. Individual’s blood pressure was measured three times using a mercury sphygmomanometer after seating for at least 10 min, the average of the three measurements was recorded for analysis.

After at least 8 h of overnight fasting, fasting blood samples were obtained in the morning and were measured on Beckman Coulter AU5800 clinical chemistry analyzer. The biochemical indicators detected including fasting plasma glucose (FPG), fructosamine, hemoglobin (HGB), hemoglobulin A1c(HbA1c), triglycerides (TG), total cholesterol (TC), red blood count (RBC), white blood count (WBC), platelet (PLT), neutrophil percentage (NEUT%), high-density lipoprotein cholesterol (HDL-C), low-density lipoprotein cholesterol (LDL-C). We also collected data on liver and renal function tests including alanine aminotransferase (ALT), alkaline phosphatase (ALP), glutamyltransferase (GGT), total protein (TP), albumin (ALB), urea, uric acid, creatinine was also measured.

### Diagnosis of MS and NAFLD

The study endpoint was the occurrence of MS, which were ascertained according to criteria jointly defined by the International Diabetes Federation (IDF) and AHA/NHLBI^36^. The presence of three or more of five components including abdominal obesity, high TG, low HDL-C, abnormal blood pressure and high FPG was determined to be MS for participants. These factors were diagnosed based on examination of hospital records by experienced endocrinologists.

The assessment and diagnosis of NAFLD was based on abdominal ultrasonography (EsaoteMyLab70). Water and food were forbidden for 12 h before examination. The ultrasonography examination was performed by two experienced radiologists who were unaware to the study. Images were captured in a standard manner with the patient in the supine position with the right arm raised above the head. To ensure the consistency, reliability and accuracy of the results, all images were inspected both by the radiologists and physicians. Fatty liver was diagnosed as brightness liver and a diffusely echogenic change in the liver parenchyna, by imaging.

### Statistical analysis

The subjects were divided into four groups according to their BMI and NAFLD status: Lean control and NAFLD, overweight/obese control and NAFLD (lean: BMI < 23 kg/m^2^, overweight/obese: BMI ≥ 23 kg/m^2^). Descriptive analyses were performed, categorical and continuous variables were analyzed using chi-square test and Welch ANOVA test, respectively. Further pairwise comparison was performed using LSD test. All data were reported as mean ± standard deviation (SD) or proportion (%) as appropriate.

We calculated person-years of follow-up, and a flexible parametric proportional hazards model was used to evaluate the association between NAFLD status at baseline and incident of MS. Kaplan–Meier survival curves were constructed to demonstrate the MS outcomes in the four groups over time. Univariate and multi variate cox regression analysis was successively performed, and the multivariable adjusted hazard ratios (HRs) for 95% confidence intervals (CIs) were calculated. Afterwards, the logistic regression analysis was carried out to assess the influence of NAFLD development and BMI changes on the MS risk. All statistical tests were two-sided, and *P* value < 0.05 was considered statistically significant. Analyses were performed using SPSS (version 18.0; Beijing Stats Data Mining Co. Ltd, Beijing, China). All the sample sizes for statistics were satisfied with the minimum sample size that calculated using PASS 2021 software (NCSS, LLC).

## Supplementary Information

Supplementary Information.

## Data Availability

The data that support the findings of this study are available from the corresponding author on reasonable request.

## Abbreviations

BMI: Body mass index
NAFLD: Non-alcoholic fatty liver disease
MS: Metabolic syndrome
WC: Waist circumference
SBP: Systolic blood pressure
DBP: Diastolic blood pressure
FPG: Fasting plasma glucose
HGB: Hemoglobin
HbA1c: Hemoglobulin A1c
TG: Triglycerides
TC: Total cholesterol
HDL-C: High-density lipoprotein cholesterol
LDL-C: Low-density lipoprotein cholesterol
RBC: Red blood cell
WBC: White blood cell
PLT: Platelet
NEUT: Neutrophil
ALT: Alanine aminotransferase
ALP: Alkaline phosphatase
GGT: Glutamyl transferase
TP: Total protein
ALB: Albumin
UA: Uric acid
FFAs: Free fatty acids
