# Supplementary material for: Lean non-alcoholic fatty liver disease (Lean-NAFLD) and the development of metabolic syndrome: a retrospective study
Source: Sci Rep. 2022 Jun 29;12:10977. doi: 10.1038/s41598-022-14701-0 (PMC9243064; doi:10.1038/s41598-022-14701-0)
Supplement: Supplementary file 1 — Supplementary Information. [file 41598_2022_14701_MOESM1_ESM.docx]

**Table S1.** Baseline demographic characteristics of male and female participants in four groups.

|  | Female | | | | *p* | Male | | | | *p* |
| --- | --- | --- | --- | --- | --- | --- | --- | --- | --- | --- |
|  | Lean non-NAFLD | Obesity/overweight non-NAFLD | Lean NAFLD | Obesity/overweight NAFLD |  | Lean non-NAFLD | Obesity/overweight non-NAFLD | Lean NAFLD | Obesity/overweight NAFLD |  |
| Participants, | 5569 | 1242 | 359 | 832 | <0.001 | 3382 | 2669 | 675 | 3667 | <0.001 |
| BMI(kg/m^2^) | 20.19±1.59 | 24.46±1.41 | 21.61±1.12 | 25.89±2.33 | <0.001 | 20.93±1.477 | 24.73±1.39 | 21.94±0.92 | 26.22±2.24 | <0.001 |
| WC (cm) | 72.27±6.70 | 84.82±6.90 | 80.51±5.61 | 91.16±7.28 | <0.001 | 76.55±7.32 | 87.42±7.28 | 84.16±6.24 | 93.30±7.91 | <0.001 |
| SBP (mmHg) | 69.53±9.25 | 73.08±9.79 | 74.27±9.60 | 76.47±9.43 | <0.001 | 74.15±9.31 | 77.1±9.489 | 77.49±9.03 | 80.16±9.65 | <0.001 |
| DBP (mmHg) | 110.21±14.40 | 118.26±15.76 | 121.93±17.25 | 127.59±17.04 | <0.001 | 120.46±14.51 | 125.83±14.02 | 124.77±14.76 | 128.87±14.46 | <0.001 |
| FPG (mmol/L) | 4.85±0.59 | 4.98±0.54 | 5.24±0.95 | 5.28±0.78 | <0.001 | 4.94±0.71 | 5.06±0.79 | 5.22±1.08 | 5.24±0.97 | <0.001 |
| Fructosamine (mmol/L) | 1.48±0.19 | 1.50±0.18 | 1.63±0.22 | 1.60±0.20 | <0.001 | 1.46±0.18 | 1.49±0.20 | 1.55±0.23 | 1.54±0.21 | <0.001 |
| HGB(g/L) | 129.4±10.87 | 129.83±11.46 | 132.56±10.49 | 133.82±10.16 | <0.001 | 151.51±10.77 | 153.07±10.33 | 153.72±10.38 | 154.41±9.98 | <0.001 |
| HbA1c (%) | 5.38±0.47 | 5.45±0.48 | 5.69±0.72 | 5.71±0.52 | <0.001 | 5.47±0.59 | 5.53±0.66 | 5.62±0.81 | 5.70±0.80 | <0.001 |
| **TG (mmol/L)** | **1.00±0.53** | **1.21±0.64** | **1.79±1.22** | **1.67±0.90** | **<0.001** | **1.19±0.65** | **1.44±1.14** | **1.88±1.30** | **1.98±1.27** | **<0.001** |
| TC (mmol/L) | 4.70±0.88 | 4.87±0.91 | 5.25±0.98 | 5.22±0.97 | <0.001 | 4.63±0.83 | 4.77±0.87 | 4.98±0.92 | 4.96±0.91 | <0.001 |
| HDL-C (mmol/L) | 1.60±0.34 | 1.47±0.31 | 1.38±0.34 | 1.36±0.29 | <0.001 | 1.39±0.33 | 1.27±0.29 | 1.26±0.31 | 1.16±0.27 | <0.001 |
| LDL-C (mmol/L) | 2.39±0.66 | 2.64±0.68 | 2.99±0.74 | 3.02±0.74 | <0.001 | 2.51±0.64 | 2.73±0.67 | 2.86±0.69 | 2.95±0.68 | <0.001 |
| RBC (10^9^/L) | 4.38±0.32 | 4.42±0.33 | 4.49±0.35 | 4.52±0.34 | <0.001 | 4.97±0.40 | 5.03±0.39 | 5.05±0.42 | 5.07±0.38 | <0.001 |
| WBC (10^9^/L) | 5.66±1.40 | 5.91±1.51 | 5.93±1.42 | 6.13±1.48 | <0.001 | 6.05±1.56 | 6.28±1.71 | 6.34±1.50 | 6.68±1.55 | <0.001 |
| PLT (10^9^/L) | 221.93±52.99 | 229.11±53.47 | 228.83±58.84 | 232.62±56.44 | <0.001 | 205.22±48.29 | 206.84±46.23 | 217.13±50.39 | 216.73±49.36 | <0.001 |
| NEUT (%) | 58.03±8.11 | 58.69±7.92 | 57.67±8.09 | 58.04±8.18 | 0.040 | 55.70±7.97 | 56.19±7.68 | 56.30±7.48 | 56.47±7.31 | <0.001 |
| **ALT(U/L)** | **16.12±16.77** | **17.85±12.22** | **26.30±21.58** | **25.75±21.15** | **<0.001** | **21.64±19.78** | **25.89±33.71** | **29.09±17.69** | **35.90±31.75** | <0.001 |
| ALP(U/L) | 60.06±16.15 | 65.91±18.06 | 73.37±19.38 | 73.78±18.62 | <0.001 | 70.02±15.60 | 69.89±15.82 | 72.48±16.68 | 72.05±16.30 | <0.001 |
| **GGT(U/L)** | **15.79±11.20** | **18.95±17.85** | **24.28±24.56** | **24.94±16.72** | **<0.001** | **24.25±17.69** | **30.04±25.00** | **38.16±28.18** | **41.48±26.226** | <0.001 |
| TP(g/L) | 74.74±3.54 | 74.32±3.56 | 75.15±3.47 | 75.19±3.39 | <0.001 | 74.32±3.56 | 74.77±3.54 | 74.64±3.54 | 75.04±3.42 | <0.001 |
| ALB(g/L) | 45.98±2.29 | 45.10±2.25 | 45.74±2.27 | 45.44±2.17 | <0.001 | 45.10±2.25 | 46.86±2.41 | 47.12±2.28 | 46.93±2.31 | <0.001 |
| Urea(mmol/L) | 4.38±1.09 | 4.47±1.12 | 4.67±1.10 | 4.79±1.19 | <0.001 | 4.47±1.12 | 5.09±1.28 | 5.04±1.14 | 5.08±1.19 | 0.382 |
| UA (μmoI/L) | 260.21±52.38 | 278.86±58.86 | 301.84±62.19 | 322.39±69.27 | <0.001 | 278.86±58.86 | 369.99±71.55 | 377.37±70.33 | 393.74±76.73 | <0.001 |
| Creatinine (μmoI/L) | 69.05±7.56 | 69.77±8.35 | 69.47±8.25 | 70.59±8.56 | <0.001 | 69.77±8.35 | 89.2±13.07 | 86.67±8.92 | 88.36±10.02 | <0.001 |


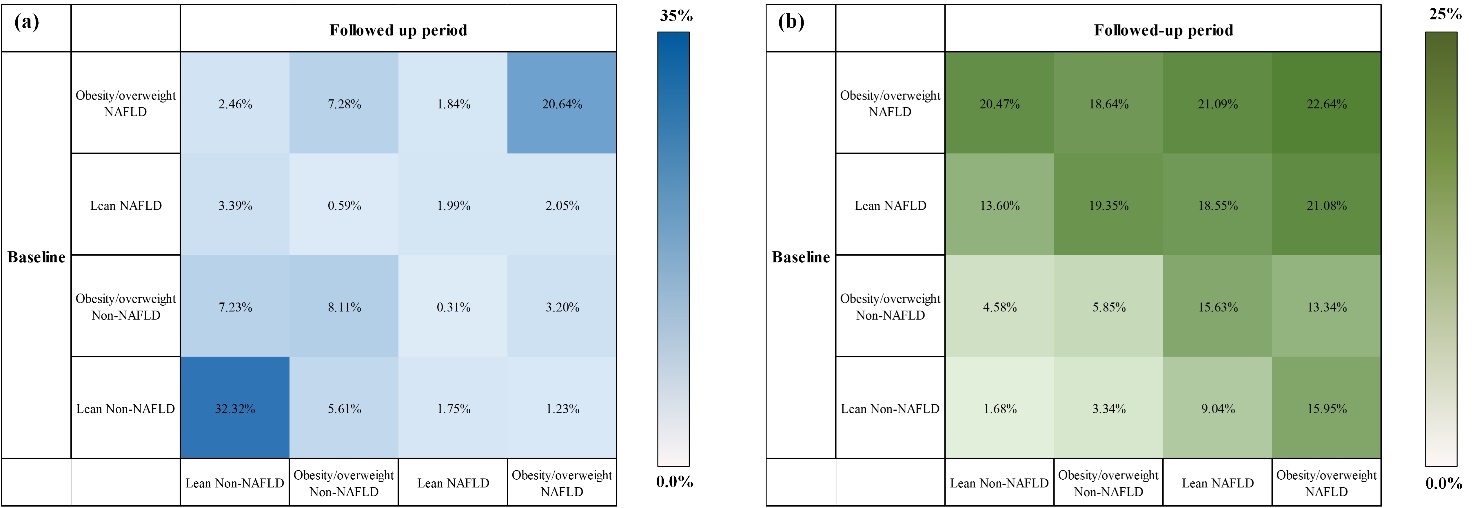


**Figure S1. (a)** The proportion of subjects with BMI and NAFLD status changes during the followed-up periods. **(b)**The incidence of MS in participants with status changes.


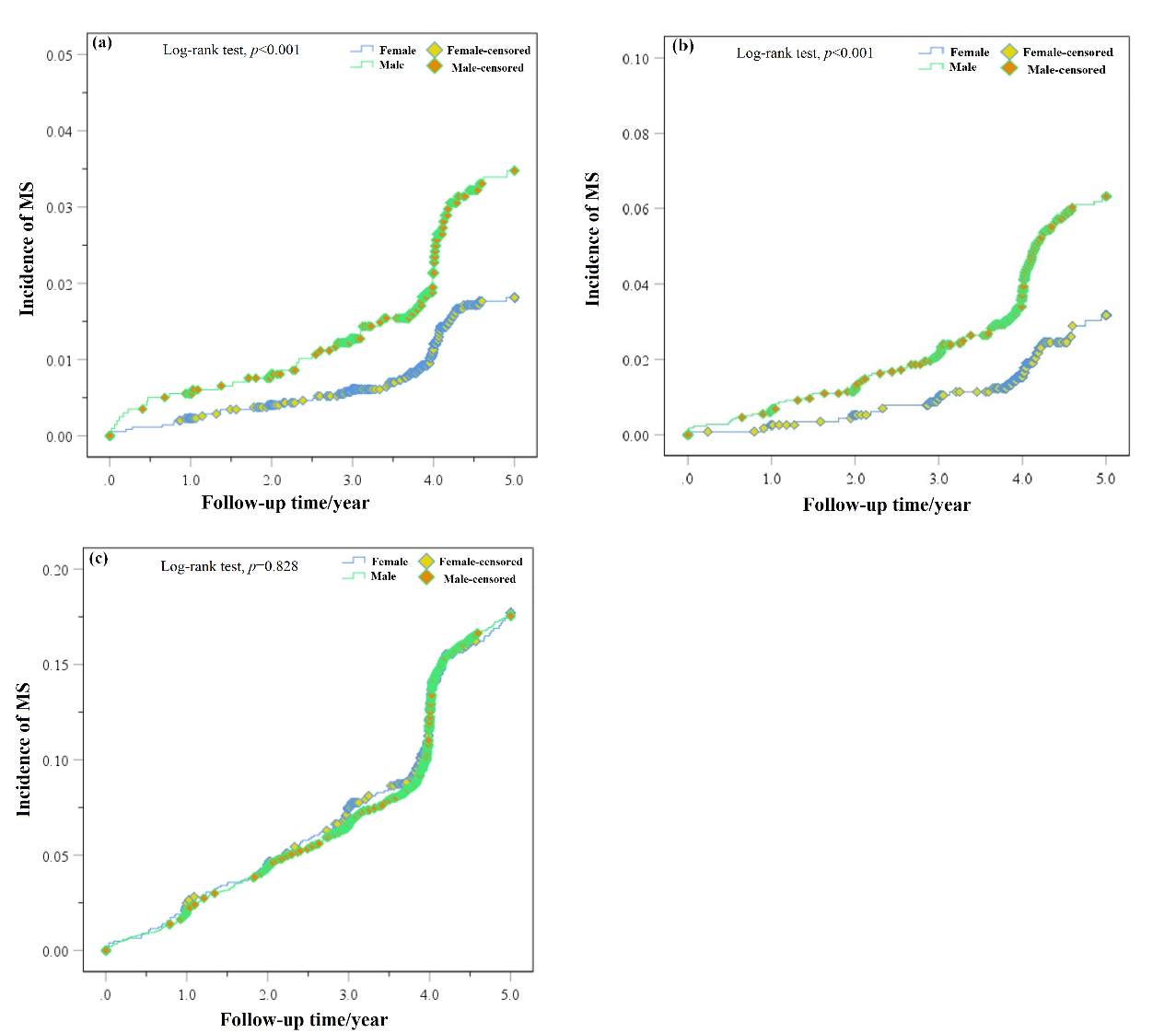


**Figure S2.** The incidence of MS in different gender after adjustment of age. (a) lean non-NAFLD, (b) obesity/overweight non-NAFLD, (c) obesity/overweight NAFLD.
